# Micropillar/Microwell Chip Assessment for Detoxification of Bisphenol A with Korean Pear (*Pyrus pyrifolia*)

**DOI:** 10.3390/mi11100922

**Published:** 2020-10-03

**Authors:** Dong Woo Lee, Moo-Yeal Lee, Sukkil Koh, Mihi Yang

**Affiliations:** 1 Department of Biomedical Engineering, Konyang University, Daejeon 35365, Korea; dw2010.lee@gmail.com; 2 Department of Chemical and Biomedical Engineering, Cleveland State University, Cleveland, OH 44115-2214, USA; m.lee68@csuohio.edu; 3 Department of Toxicology, College of Pharmacy, Sookmyung Women’s University, Seoul 04310, Korea; srosekoh@gmail.com

**Keywords:** bisphenol A, 3D cell culture chip, Korean pear, alternative animal study, detoxification

## Abstract

A micropillar/microwell chip platform with 3D cultured liver cells has been used for HTP screening of hepatotoxicity of bisphenol A (BPA), an endocrine-disrupting chemical. We previously found the hepatotoxicity of BPA is alleviated by alcohol dehydrogenase (ADH) and aldehyde dehydrogenase 2 (ALDH2). In this study, we have tested potential BPA detoxification with Korean pear (*Pyrus pyrifolia*) extract, stimulators of ADH and ALDH, as well as arbutin, a reference compound in the pears, on the micropillar/microwell chip platform with human liver cells. Surprisingly, the toxicity of BPA was reduced in the presence of Korean pear extract, indicated by significantly increased IC_50_ values. The IC_50_ value of BPA with Korean pear extract tested against HepG2 cells was shifted from 151 to 451 μM, whereas those tested against Hep3B cells was shifted from 110 to 204 μM. Among the tested various concentrations, 1.25, 2.5, and 5 mg/mL of the extract significantly reduced BPA toxicity (*P*s < 0.05). However, there was no such detoxification effects with arbutin. This result was supported by changes in protein levels of ADH in the liver cells.

## 1. Introduction

For the safety of new drugs and risk assessment of potential environmental toxicants, alternative animal studies have been developed for the purpose of 3R, also known as replacement—the substitution of conscious living higher animals for insentient material; reduction—reduction in the number of animals used to obtain information of a given amount and precision; refinement—any decrease in the severity of inhumane procedures applied to those animals [1]. To test a daunting number of xenobiotics, including drugs, in various experimental conditions, the alternative animal platforms should be biomimetic and high-throughput (HTP). 

Nonetheless, metabolism-induced toxicity of xenobiotics in the liver has been overlooked, which could be critical to determine potential human toxicity due to the significant discrepancy between animal and human metabolism in the liver. Many compounds that appear promising in preclinical species, fail in human clinical trials due to safety concerns [2]. In fact, metabolism is a double-sided knife, as xenobiotics can be biotransformed into inactive or bioactive intermediates by metabolic enzymes. The reactive electrophilic intermediates bind with targeted nucleophiles of cellular components, including cysteine, sulfhydryl, lysine, thiolate and histidine amino residues in proteins, guanine and adenine in DNA, and hydroxyl, carboxyl, and phosphate in lipids molecules, etc. [3], and induce various toxic responses, including cancers [4]. Thus, many toxicologists have been longing for alternative animal platforms that could accurately mimic compound metabolism in the human liver and determine potential augmented toxicity and detoxification of compounds. To this end, our group has been developing 3D cell-based HTS platforms, including micropillar/microwell chips, to determine metabolism-induced toxicity of compounds [5,6,7,8,9,10]. 

Using the micropillar/microwell chip platform with 3D cultured liver cells, we have simultaneously tested over 500 conditions with different materials, concentrations, and metabolic enzymes and screened the toxicity of bisphenol A (BPA), an endocrine-disrupting chemical [7]. BPA, 2,2-bis (4-hydroxyphenyl) propane, was synthesized at the end of the nineteenth century and has a similar structure with diethylstilbestrol (DES), a synthetic estrogen drug, which has been banned due to its carcinogenicity [11]. Unlike DES, BPA has been used widely in industry for the production of plastics. However, BPA has been considered as an endocrine-disrupting chemical for over three decades [12]. Although the toxicity of BPA in humans is still a matter of debate, people are easily exposed to BPA in daily lives due to a broad usage of BPA-derived plastics, including polycarbonates, epoxy resins, and some polysulfones, for plastic bottles, storage containers, water pipes, and coatings in many food and beverage cans [13]. Therefore, monitoring the biological impact of BPA on human health and its detoxification from unavoidable exposure have to be investigated. To understand detoxification pathways of BPA, detailed investigation of BPA metabolism is necessary. When administered orally in humans, BPA is mostly converted into glucuronide metabolites by phase II metabolizing enzymes, such as UDP-glucuronosyltransferases (UGTs), and eliminated in urine [14]. As demonstrated by our previous DataChip/MetaChip study, BPA toxicity could be alleviated by alcohol dehydrogenase (ADH), aldehyde dehydrogenase 2 (ALDH2), and sulfotransferase 1E1 (SULT1E1), whereas it could be augmented by cytochrome P450 2E1 (CYP2E1), all tested on the micropillar/microwell chip [7]. In this study, we tested Korean pear extract that could potentially induce or stimulate the above enzymes, leading to BPA detoxification. 

Korean pear (*Pyrus pyrifolia cv. Shingo*) has been used as a traditional medicine for alleviating an alcohol hangover in Korea and its extract has been known to stimulate both ADH and ALDH activities by over 2- and 1.3-fold, respectively [15]. In addition, Korean pears have plenty of polyphenols, including arbutin, which could be potential active ingredients to stimulate ADH and ALDH activities. Therefore, we tested Korean pear extract and arbutin as potential materials for BPA detoxification on the micropillar/microwell chip with human liver cells.

## 2. Materials and Methods

### 2.1. Materials

BPA and other chemicals in analytical grade were obtained from Sigma-Aldrich Korea (Yongin-si, Gyeonggi-do, Korea). Korean pears were kindly donated from the Pear Research Institute (Naju, Jeollanam-do, Korea). After washing the pears with water, they were dried and cut into 6–8 pieces. After grinding and juicing the pear pieces, 615 mL of the pear juice was centrifuged at 13,226g for 15 min. The supernatant (600 mL) was concentrated under reduced pressure using a rotary evaporator (EYELA, N-1110, Tokyo, Japan) into 90.6 g. The concentrated pear extract was further freeze-dried (Operon, FDUT-8606, Gimpo-si, Gyeonggi-do, Korea) into 82.8 g, which contained 24 brix, i.e., 24% of sugar, 882 μg/g of arbutin, and 50.7 μg/g of chlorogenic acid.

### 2.2. Cell Culture and Preparation

Human hepatoma cell lines, including Hep3B and HepG2 cells, were obtained from American Type Culture Collection (ATCC, Manassas, VA, USA) and grown in T-75 cell-culture flasks with Dulbecco’s Modified Eagle Medium (DMEM) supplemented with 10% fetal bovine serum (Sigma-Aldrich Korea) and 1% Penicillin–Streptomycin (Thermo Fisher Scientific Korea, Seoul, Korea) in a humidified 5% CO_2_ incubator (Thermo Fisher Scientific Korea) at 37 °C. Cell suspension was prepared by trypsinization of a confluent layer of the cells with 0.6 mL of 0.05% trypsin–0.53 mM EDTA (Thermo Fisher Scientific Korea) and mixing with 7 mL of 10% FBS-supplemented DMEM. After centrifugation at 176 g for 3 min and removing the supernatant, the cell pellet was resuspended with 10% FBS-supplemented DMEM to a final concentration of 4 × 10^6^ cells/mL.

To culture Hep3B and HepG2 cells in 3D on the micropillar/microwell chip platform, 500 μL of each cell suspension at 4 × 10^6^ cells/mL was mixed with 250 μL of 3% alginate in distilled water and 250 μL of DMEM to obtain a final concentration of 2 × 10^6^ cells/mL in 0.75% alginate.

### 2.3. Preparation of the Micropillar Chip with Cells and the Microwell Chip with Compounds

**Figure 1:**
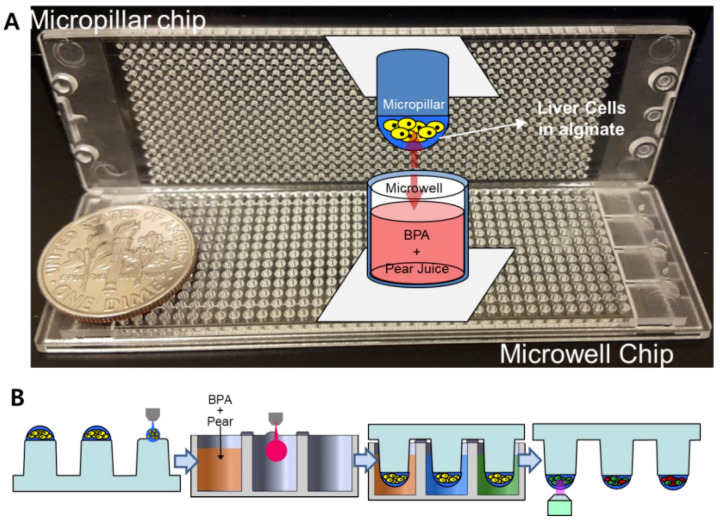
Assessment of detoxification mechanisms of Korean pear extract or arbutin on bisphenol A (BPA) with the micropillar/microwell chip platform: (**A**) Picture of injection-molded micropillar and microwell chips. (**B**) Schematic of experimental procedures for high-throughput screening of pear extracts or arbutin with BPA.

**Figure 2:**
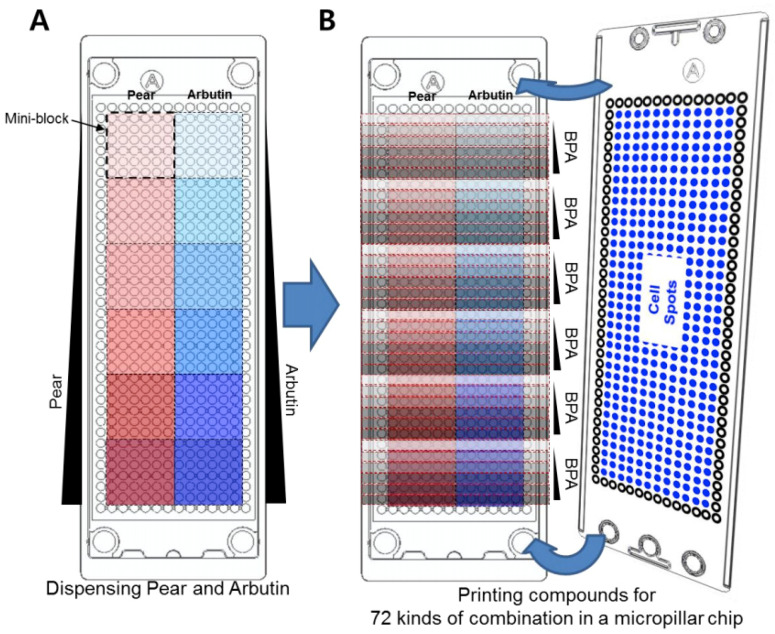
Layout of compound printing in the microwell chip to generate 72 combinations of compound test conditions in 12 mini-blocks: (**A**) Printing of six different concentrations of Korean pear extract and arbutin in twelve mini-blocks in the microwell chip (one concentration of pear extract or arbutin per mini-block). (**B**) Subsequent printing of six different concentrations of BPA in each mini-block in the microwell chip, followed by sandwiching the micropillar chip with human liver cells (Hep3B or HepG2 human hepatoma cell lines) in alginate.

For high-throughput 3D cell-based assays, a micropillar chip and a complementary microwell chip manufactured via injection molding of polystyrene by Medical and Bio Device Korea (MBD Korea, Suwon, Korea) were used (Figure 1A) [10]. Approximately 50 nL of the cell–alginate mixture was printed on the micropillar chip using ASFA^™^ microarray spotter (MBD Korea) to achieve 100 cells in 0.75% alginate per micropillar. After 1–2 minutes of alginate gelation on a chilling deck at 4 °C, the micropillar chip containing the cells was sandwiched with the microwell chip containing 950 nL of DMEM overnight prior to compound exposure. To perform combinatorial BPA and pear extract/arbutin tests against the liver cell types, five different concentrations of Korean pear extract (1.2, 3.7, 11.1, 33.3, and 100 mg/mL) and arbutin (1.2, 3.7, 11.1, 33.3, and 100 μM) as well as no material control were dispensed into twelve mini-blocks, with a single concentration in each block consisting of six by six microwells (Figure 2A). This was followed by dispensing five different concentrations of BPA (50, 100, 200, 400, and 800 μM) and no BPA control into each mini-block consecutively (Figure 2B). Thus, the microwell chip contained BPA alone, Korean pear extract alone, arbutin alone, and combinations of BPA and Korean pear extract or arbutin (total 72 conditions per microwell chip tested) at 2-fold diluted final concentrations.

### 2.4. Compound Exposure, Cell Staining, and Data Analysis 

The micropillar chip with either Hep3B or HepG2 cells in alginate was sandwiched with the microwell chip containing test materials, and the sandwiched chips were placed in a humid chamber and incubated in the 5% CO_2_ incubator at 37 °C for 4 days. After incubation, the micropillar chip was separated, rinsed with PBS solution twice, and stained with 4mM of calcein AM. An automated fluorescence microscope (S^+^ Chip Scanner, MBD Korea) was used to obtain green fluorescent cell images from the micropillar chip and acquire the intensity and area of cell spots. For intensity analysis, image analysis software (S^+^ Chip Analyzer, MBD Korea) defined the boundary of cell spots and calculated mean intensity by dividing total green fluorescence intensity (8 bit green code among RGB code: 0–255) on each micropillar. For area analysis, the software counted the number of pixels, compared to the background (30 green code), and calculated the area of the 3D cultured colonies. To calculate IC_50_ values, the percentage of live cells at different concentrations was calculated with the intensity and the area. Percent cell viability was calculated by normalization with those from no compound condition (100% viability). The sigmoidal dose–response curves (variable slope) and IC_50_ values (i.e., concentration of the compound, where 50% of cell growth was inhibited) were obtained using the following equation:

(1) $$Y=Bottom+\left[\frac{Top-Bottom}{1+10^{(\log\mathrm{IC}50-X)\times H}}\right]$$

where IC_50_ is the midpoint of the curve, *H* is the hill slope, *X* is the logarithm of compound concentration, and *Y* is percent cell viability, starting at *Bottom* (0%) and going to *Top* (100%) with a sigmoid shape. To analyze statistical difference in IC_50_ values in the presence and absence of BPA, pear extract, arbutin, and their combinations, p values were calculated with one-way ANOVA (Analysis of Variance). All statistical analyses were done with JMP version 4 (SAS Institute, Cary, NC, USA). Statistical significance was defined as *p* < 0.05.

### 2.5. Western Blot Analyses

To analyze changes in the expression level of ADH after exposing HepG2 cells to no compound (control), 100 μM BPA, and a mixture of 100 μM BPA and 2.5 mg/mL pear extract, we performed Western blot analysis. Total HepG2 cell lysates were prepared with cOmplete™ Lysis-M buffer solution (Roche Life Science, Penzberg, Germany). Protein extracts were resolved by 4–20% Mini-PROTEAN TGX™ Precast Protein Gels (Bio-Rad, Hercules, CA, USA) and transferred onto iBlot^®^ PVDF gel Transfer Stacks (Thermo Fisher Scientific Korea). After blocking non-specific binding sites for 1 h in 5% bovine serum albumin in Tris-buffered saline containing 0.1% Tween-20 (TBST), each membrane was incubated overnight at 4 °C with specific primary antibodies. Following manufacturer’s instructions, we used ADH antibody (1:1000, Abcam, Cambridge, UK) and anti-beta actin (1:2000, Abcam). The membranes were washed in TBST and incubated further with horseradish peroxidase (HRP)-conjugated anti-rabbit secondary antibodies (1:5000, Abcam) at room temperature. Protein bands were detected using an enhanced ECL^™^ Western blotting detection kit (GE Healthcare, Little Chalfont, UK).

## 3. Results and Discussion

### 3.1. Cytotoxicity of BPA, Korean Pear Extract, and Arbutin

**Figure 3:**
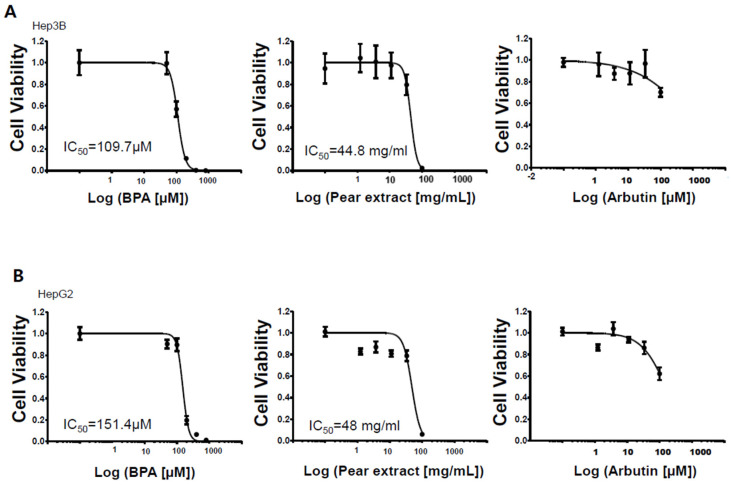
Dose response curves of BPA, Korean pear extract, and arbutin against human liver cells: (**A**) Hep3B cells and (**B**) HepG2 cells.

Before evaluating the effects of pear extract and arbutin on BPA-induced cytotoxicity, the toxicity of individual materials was tested against Hep3B and HepG2 cells (Figure 3). BPA and pear extract were toxic to Hep3B and HepG2 cells at the highest concentrations, whereas arbutin was nontoxic at the test concentrations. For the pear extract, a high concentration of sugar in the extract can cause cell cytotoxicity via increased osmolality [11]. IC_50_ of the pear extract, 44 mg/mL, included 24 % of sugar, 10.56 mg/mL. The IC_50_ values obtained from BPA and Korean pear extract were 110 μM and 45 mg/mL, respectively, on Hep3B cells. In addition, those obtained on HepG2 cells were 151 μM and 48 mg/mL, respectively. There was no significant difference in IC_50_ values from Hep3B and HepG2 cells. We are quite unsure which ingredients make Korean pear extract toxic against Hep3B and HepG2 cells.

### 3.2. Changes in BPA Toxicity in the Presence of Korean Pear Extract or Arbutin

**Figure 4:**
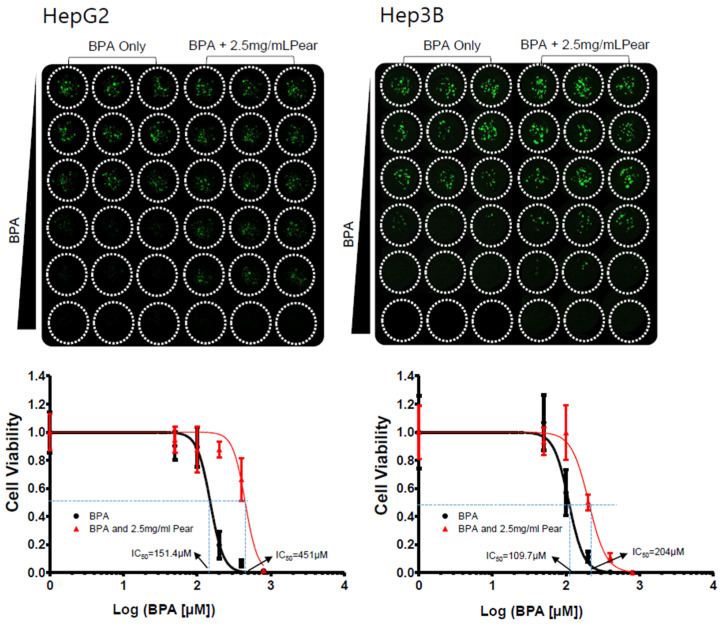
Korean pears reduced BPA cytotoxicity in both Hep3B cells and HepG2 cells under the micropillar/microwell platform.

**Figure 5:**
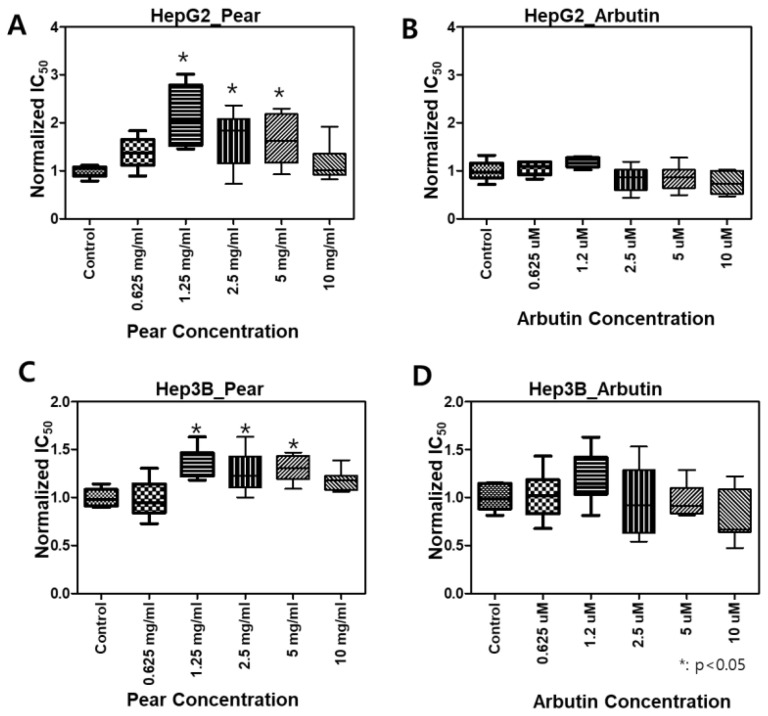
Not arbutin but Korean pears increased normalized IC_50_ values of BPA: *(*P* < 0.05), compared to the control (BPA only treated); *N* = 3 for each group.

**Figure 6:**
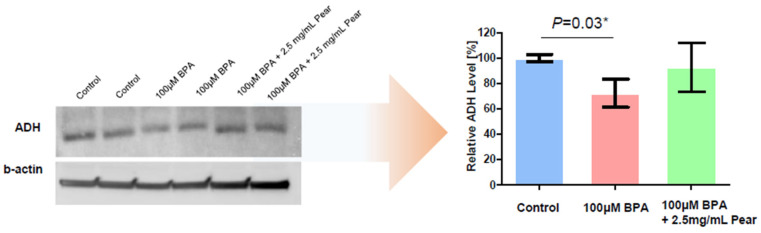
Comparison of alcohol dehydrogenase (ADH) protein expression among no compound (control), 100 μM BPA, and a mixture of 100 μM of BPA and 2.5 mg/mL of pear extract.

To evaluate BPA detoxification in the presence of Korean pear extract and arbutin, we considered to use the maximum concentration of 10 mg/mL pear extract and 10 μM arbutin, where the baseline cell viability was greater than 80% at those concentrations (Figure 3). The combination effects were tested with varying concentrations of BPA as well as pear extract and arbutin. Surprisingly, BPA toxicity was significantly reduced in the presence of 2.5 mg/mL Korean pear extract against both Hep3B and HepG2 cells (Figure 4). The IC_50_ value of BPA with Korean pear extract tested against HepG2 cells was shifted from 151 to 451 μM, whereas those tested against Hep3B cells was shifted from 110 to 204 μM. A similar trend of BPA detoxification was obtained in the presence of 1.25, 2.5, and 5 mg/mL Korean pear extract, whereas there was no significant BPA detoxification observed in the presence of arbutin (Figure 5). To better understand these surprising results, we performed Western blot analysis of ADH expression after exposing HepG2 cells to no compound (control), 100 μM BPA, and a mixture of 100 μM BPA and 2.5 mg/mL pear extract. As a result, the expression level of ADH was reduced after BPA exposure, but maintained in the presence of Korean pear extract (Figure 6). In our previous study, ADH played a significant role in alleviating BPA toxicity [7]; therefore, the present study suggests Korean pears reduced BPA toxicity via ADH stimulation.

We encapsulated two types of human liver cells in alginate on the micropillar chip to simulate hepatotoxicity of BPA and its combination with Korean pear extract and arbutin and meet the need for HTS of compounds in 3D-cultured cells. The micropillar chip facilitated 3D cell-based assays at a hundreds-times smaller scale (100 cells in 50 nL) as compared to that in more traditional 2D cell-based assays performed in microtiter well plates (10,000 cells in 200 µL) [16]. In addition, the present hepatic cell microarrays provide more physiologically relevant and robust 3D cell culture for toxicity analyses, unlike traditional 2D monolayer cultures in 96-well plates [17]. For comparison of 22 drugs including acetaminophen, the IC_50_ values obtained from our 3D chip platform were correlated with rat LD_50_ values, human C_max_ values, and drug-induced liver injury categories to predict adverse drug reactions in vivo [5,6]. Therefore, our chip platform could be used as an HTP early-stage, microscale alternative to conventional in vitro multi-well plate platforms and provide a rapid and inexpensive assessment of cytotoxicity for safety assessment. Recently, the present micropillar/microwell platform can be applicable as a cancer spheroid array chip for selecting effective drugs [18].

Some materials, such as luteolin-supplemented diets and multi-strain probiotics, showed to ameliorate toxic effects of BPA in vitro and in vivo studies via anti-oxidative stress and anti-inflammation mechanisms [19,20]. In the present study, we evaluated BPA detoxification with the materials that could stimulate metabolic enzymes. Using various phase I and II metabolizing enzymes on the micropillar/microwell chip platform, metabolic pathways of BPA via CYP2E1, SULT1E1, ADH, and ALDH2 have been evaluated in vitro [7]. It was the first report which showed involvement of ADH and ALDH2 for BPA detoxification. Thus, our present study provides new chemopreventive materials via the micropillar chip with human liver cells coupled with the microwell chip. The chip is useful to assess potential mechanisms of BPA detoxification rapidly.

Various polyphenols in pears, including arbutin, chlorogenic acid, and triterpenoids, including ursolic acid, have been reported [21]. Our results showed arbutin was not toxic in the given concentrations (≤100uM; Figure 3) and did not alleviate BPA toxicity (Figure 5). Interestingly, the pear extract showed toxicity at the highest concentration (Figure 3), 100uM, while it detoxified BPA hepatotoxicity (Figure 5). Therefore, some active compounds rather than arbutin may alleviate BPA toxicity. In East Asia, including Korea, Japan, and China, pears have been used for diverse medicinal applications, e.g., respiratory symptoms relievers, fever managements, inflammation treatment, alcohol hangover, etc., for over a millennium [22,23]. Modern scholars have testified the efficacy of pears and reported many pieces of scientific evidence to support this ancient functional food [24,25]. However, the pears have been avoided for pregnant women or stab wounds [22]. Therefore, we plan on isolating various compounds in Korean pear extracts using prep-HPLC and testing on the micropillar/microwell chip platform to find out potential active or toxic ingredients in the pears.

BPA has been recently testified with diverse in vitro studies, such as cell division or stem cell differentiation, which are based on its various endocrine disrupting mechanisms, and showed disrupted mitotic progression via disturbing spindle attachment to kinetochore and centriole duplication in cancer cell lines [26] and suppressed myogenic differentiation through the inhibition of *Akt* signaling [27]. Therefore, the present metabolism-based tests as well as the above in vitro studies can be applicable tools to screen or find out chemopreventive materials against BPA toxicity.

In addition, Korean pears have been used as traditional medicine for respiratory inflammation [22]. For novel severe acute respiratory syndrome (SARS) coronavirus-2 (SARS-CoV-2), BPA showed to affect SARS-CoV-2- infection mediators, such as angiotensin-converting enzyme 2 (ACE2) [28] and transmembrane serine protease 2 (TMPRSS2) [29], a major driver of prostate carcinogenesis [30]. Therefore, BPA and Korean pears have potential as a risk and protection for COVID-19, respectively. In the near future, the effects of Korean pears on molecular modulation of ACE2 or TMPRSS2 should be studied. In the near future, more advanced approaches, such as a combination chip with liver and gut models [31] and the modified micropillar/microwell chip platform with ACE2 or TMPRSS2 expressing cells, can realize systemic or integrated responses to “BPA and Korean pears” in the whole body and clarify their effects on SARS-CoV-2.

## 4. Conclusions

We demonstrated that the micropillar/microwell chip platform is useful to evaluate the hepatotoxicity of BPA and assess potential BPA detoxification with Korean pear extract and arbutin. We found that the toxicity of BPA is alleviated in the presence of Korean pear extract potentially due to stimulation of ADH in human liver cells. The arbutin abundant in pear skins was ineffective in BPA detoxification.
